# Differences in physician electronic health record use by telemedicine intensity: evidence from 2 academic medical centers

**DOI:** 10.1093/jamia/ocaf122

**Published:** 2025-07-11

**Authors:** Seunghwan Kim, Robert Thombley, Elise Eiden, Sunny Lou, Julia Adler-Milstein, Thomas Kannampallil, A Jay Holmgren

**Affiliations:** Roy and Diana Vagelos Division of Biology and Biomedical Sciences, Washington University School of Medicine in St Louis, Saint Louis, MO 63110, United States; Institute for Informatics, Data Science, and Biostatistics (I2DB), Washington University School of Medicine in St Louis, Saint Louis, MO 63110, United States; Division of Clinical Informatics and Digital Transformation, Department of Medicine, University of California San Francisco, San Francisco, CA 94131, United States; Department of Anesthesiology, Washington University School of Medicine in St Louis, Saint Louis, MO 63110, United States; Institute for Informatics, Data Science, and Biostatistics (I2DB), Washington University School of Medicine in St Louis, Saint Louis, MO 63110, United States; Department of Anesthesiology, Washington University School of Medicine in St Louis, Saint Louis, MO 63110, United States; Division of Clinical Informatics and Digital Transformation, Department of Medicine, University of California San Francisco, San Francisco, CA 94131, United States; Institute for Informatics, Data Science, and Biostatistics (I2DB), Washington University School of Medicine in St Louis, Saint Louis, MO 63110, United States; Department of Anesthesiology, Washington University School of Medicine in St Louis, Saint Louis, MO 63110, United States; Division of Clinical Informatics and Digital Transformation, Department of Medicine, University of California San Francisco, San Francisco, CA 94131, United States

**Keywords:** telemedicine, electronic health records, EHR metadata, audit logs, physician workflow

## Abstract

**Objective:**

Evaluate the association between telemedicine intensity and ambulatory physician electronic health record (EHR) use following the COVID-19 pandemic.

**Materials and Methods:**

This retrospective study included ambulatory physicians in 11 specialties at 2 large academic medical centers (Washington University in St Louis [WashU], University of California San Francisco [UCSF]). EHR use measures, including time-based and frequency-based, were analyzed in the post-COVID-19 period (March 1, 2021, through March 7, 2022). Multivariable regression models with 2-way fixed effects were used to assess the association between telemedicine intensity and EHR use.

**Results:**

Fully telemedicine physician-weeks were associated with higher EHR (hours per 8 patient scheduled hours; β = 3.2 at WashU, β = 1.4 at UCSF; *P* < .001) and documentation time (β = 2.7 at WashU, β = 1.4 at UCSF; *P* < .001). Several differences in discrete EHR-based tasks were observed: fully telemedicine physician-days were associated with lesser ordering, and there were mixed patterns for information seeking and clinical communication tasks.

**Discussion:**

Expanded use of telemedicine was associated with significant changes in physician EHR use post-COVID-19 onset. Increased EHR time may suggest a shift in workload, whereas decreased ordering may suggest constraints in virtual care, such as ability to perform physical examination and the reliance on patient-reported symptoms. Institutional differences usage patterns suggest that telemedicine’s impact is context-specific and provides opportunities for understanding how to optimize EHRs to support telemedicine.

**Conclusion:**

Telemedicine shifts physician EHR. Supporting physicians through optimized EHR tools, tailored workflows, and team-based interventions is essential for sustainable virtual care delivery without exacerbating EHR burden.

## Introduction

A long-term impact of COVID-19 was the expanded use of telemedicine to deliver ambulatory care.^1–5^ One year after the onset of COVID-19, half of Medicare primary care encounters were delivered via telemedicine.^6^^,^^7^ This transformation was enabled by a combination of regulatory changes and rapid adoption of new technical capabilities.^8^ Although telemedicine affords flexible access, it may also change physician work practices, including the format, structure, management, and delivery of care.^3^^,^^9–12^ Such changes may affect clinician workflows^13^: eg, telemedicine limit physicians’ ability to collect vital signs or perform physical exams for diagnostic assessment and decision making. Similarly, virtual interactions may limit assessment of physical appearance and body language.^14^

These limitations may contribute to diagnostic uncertainty, resulting in an increased reliance on the electronic health record (EHR) to access historical data (ie, increased information seeking), the need for follow-up communication, and additional diagnostic investigations (eg, more laboratory tests), or therapeutic strategies (eg, more medication ordering). Although recent work highlighted the association of telemedicine with EHR time,^15^ we know very little about the underlying patterns of EHR use, especially those related to discrete EHR functions and activities, that drives that finding. Closing this evidence gap is critical to developing a sustainable approach to incorporating telemedicine into physician work. For example, there are open questions regarding how physicians’ workdays should be structured: all telemedicine, all face-to-face, or a combination of both modalities. Understanding how these care delivery models influence physicians’ EHR use is essential to identifying potential inefficiencies in clinical workflow.

It may be that telemedicine expands the scope of EHR work or makes EHR use less efficient, due to workflow fragmentation and switching costs associated with delivering multiple modalities of care. In other words, if telemedicine encounters lead to longer EHR interaction times or increased cognitive effort, this highlights the need for optimizing EHR tools for virtual care delivery. By addressing these gaps, we can inform strategies to redesign EHR systems, optimize clinical workflows, and guide policies that ensure equitable and sustainable integration of telemedicine into clinical practice.

## Objective

We assessed 3 research questions related to the expanded use of telemedicine has shifted ambulatory physician EHR use at 2 large health systems. First, how did physician EHR use change since the onset of the COVID-19 pandemic? Second, did the time spent on the EHR, and the distribution of that time overall and outside of clinic hours, vary depending on the intensity of telemedicine encounters provided on each workday? Finally, how was telemedicine use associated with the use of specific EHR functions related to information seeking, clinical communication, and ordering intensity? Addressing these questions will help guide streamline workflow, technology, and policy efforts to design optimal approaches to ensure sustainable integration of telemedicine into ambulatory care.

## Materials and methods

### Study setting, sample, and data

We used data from ambulatory physicians practicing at: (1) Washington University School of Medicine and BJC HealthCare in St Louis, MO (WashU), and (2) University of California San Francisco (UCSF) Health in San Francisco, CA. Study sample included attending physicians across 11 ambulatory specialties at both sites: cardiology, dermatology, family medicine, general surgery, hematology and oncology, internal medicine, nephrology, neurology, obstetrics and gynecology, otolaryngology, and pediatrics. Both institutions used an Epic EHR during the study period. This study was approved by the institutional review boards at both institutions with a waiver of informed consent.

The study periods included one year before the COVID-19 pandemic (February 25, 2019, through March 1, 2020; “pre-COVID”) and one year following the pandemic (March 1, 2021, through March 7, 2022; “post-COVID onset”). We chose March 2021 as our second period to account for a 1-year washout after the start of the pandemic when face-to-face ambulatory care delivery was not consistently available.

For the study periods, we identified all scheduled, non-cancelled, billable ambulatory patient encounters. From this encounter-level data, we identified a set of days—defined as the 24-hour work period spanning 3:00 am on a calendar day through 2:59:59 am of the following calendar day—where an attending physician had 1 or more encounters (henceforth referred to as “physician-days”). We included only those physician-days occurring during calendar weeks where the physician had at least 4 hours of patient scheduled time.

Discrete clinician EHR activities were captured using Epic audit log data,^16^ retrieved from the ACCESS_LOG tables in Epic’s Clarity database. Additional information variables extracted included proportion of evaluation & management (E/M) encounters (CPT codes: 99201, 99202, 99203, 99204, 99205, 99211, 99212, 99213, 99214, 99215), percentage of level 4 or 5 E/M encounters, mean appointment length, total number of scheduled appointments, percentage of inpatient encounters, and percentage of notes written with a human scribe or other team-member. We combined these 2 data sources and performed analysis at both the physician-day level and physician-week levels.

### Measures

#### Telemedicine intensity

The primary *independent* variable was the daily proportion of a physician’s completed ambulatory encounters that were conducted via telemedicine. This measure of telemedicine intensity was calculated by dividing the number of completed synchronous video and audio-only telemedicine encounters by the total number of completed ambulatory encounters, excluding unscheduled telephone calls and scheduled visits that were not completed. Days were categorized into 4 categories: entirely face-to-face, entirely telemedicine, mixed modality days with 50% or fewer telemedicine visits (0< and ≤50%; henceforth referred to as “≤50% mixed telemedicine”), and mixed modality days with greater than 50% telemedicine visits (50%< and <100%; henceforth referred to as “>50% mixed telemedicine”). Weekly telemedicine encounter proportions were also calculated and categorized in the same way.

#### Time-based outcomes

We defined 2 types of EHR use measures for our *dependent* variables: time-based at the physician-week level and frequency-based at the physician-day level.^17^ Time-based measures included total time spent on the EHR (EHR-Time_8_), time spent on the EHR outside of regular working hours (WOW_8_), total time spent documenting clinical notes and the time spent on clinical notes outside of regular working hours (Note-Time_8_, WOW-Note-Time_8_), and EHR time spent on mobile devices (MOB_8_). We constructed time-based EHR measures using timestamps of actions collected from the EHR-based audit log events bounded by the time when users logged in and out of the EHR. A 1-minute timeout criteria was defined (ie, time stopped 1 minute after the last recorded EHR action) to denote periods of inactivity, which were excluded from the calculation, as used in prior studies.^15^

To facilitate comparisons across different clinical volumes, all time-based measures were normalized to 8 patient scheduled hours (PSHs) standard. PSHs were calculated as the sum of all scheduled appointment durations on each physician-day and physician-week. Time-based measures that were categorized as “outside of working hours” were based on the work performed between 6:00 pm and 3:00 am.^15^ Time spent on clinical notes was calculated separately using metadata-defined note start and end times and added as a continuous block to total EHR time, independent of audit log activity.

#### Frequency-based outcomes

We created 15 frequency-based measures designed to capture 3 broad categories of EHR activities: (1) information seeking, (2) ordering intensity, and (3) clinical communication. Measures were calculated for all included physician-days with 1 or more scheduled outpatient encounters.

We created 5 *information seeking measures*: (1) total count of patient record review actions of any outside information for any patient (eg, Epic Care Everywhere; “Outside record views”); (2) count of distinct patients whose outside records were viewed (“Patients with outside record views”); (3) count of distinct patient encounters viewed by the physician (“Viewed encounters”); (4) count of distinct patient encounters edited by the physician (“Edited encounters”); and (5) total count of chart review actions (“Chart review actions”).

We created 4 measures of *ordering intensity*: (1) total count of order sessions (signing, deleting or otherwise modifying 1 or more orders for the same patient; “Ordering sessions”); (2) total count of changes to the patients’ medication lists (“Medication list changes”); (3) total count of medication orders placed (“Medication orders”); (4) count of distinct patients with any changes to their medication lists (“Patients with medication orders”).

We created 6 measures of *clinical communication* focused on asynchronous messaging: 4 were focused on patient-specific messaging (eg, MyChart messages) and 2 focused on overall asynchronous messaging volume. Patient-specific messaging measures were: (1) total count of patient-specific messages sent by a physician (“Patient-message sent”); (2) count of distinct patients to which a physician sent 1 or more patient-specific messages (“Patients with a patient-message sent”); (3) total count of patient-specific messages viewed by a physician (“Patient-messages viewed”); (4) count of distinct patients for which the physician viewed 1 or more patient-specific messages (“Patients with a patient-message viewed”). Total asynchronous messaging activities were quantified by: (1) total count of messages created by a physician (“In Basket messages created”) and (2) total count of messages viewed by a physician (“In Basket messages viewed”). Measures were aggregated to the physician-day level and were calculated by counting the number of audit log actions from each category. An overview of frequency-based measures across the 3 categories is included in the Table SA (breakdown of frequency-based EHR usage measures by category).

### Statistical analyses

We first calculated descriptive characteristics in the post-COVID onset period. Next, to address the first research question, we measured the unadjusted time-based (physician-week level) and frequency-based (physician-day level) EHR use between pre-COVID and post-COVID onset periods.

To address the second and third research questions, in the post-COVID onset period we assessed unadjusted and adjusted relationships between telemedicine intensity and each EHR use measure. We focused on the post-COVID onset period as telemedicine was not widely available during the pre-COVID period at both institutions.

In adjusted analyses, we used multivariable regression with 2-way fixed effects to examine associations between telemedicine visit intensity and physician EHR use. Each model had a time- or frequency-based EHR use measure as the dependent variable. Models included physician-level fixed effects to control for unobserved time-invariant confounders (eg, time in practice, EHR skill) that could influence EHR use. Week-level fixed effects were included to adjust for temporal trends in EHR use such as seasonal variation (eg, lower message volume and EHR time during holidays), secular changes over time, and COVID-19 infection rates. We controlled for additional time-varying covariates to account for differences in workload and patient complexity including the number of scheduled ambulatory encounters, mean length of scheduled encounters (minutes), total scheduled patient care hours, percentage of notes with help from human scribes or other team members, percentage of evaluation and management (E/M) encounters, percentage of high-complexity (level 4/5) E/M encounters, and percentage of inpatient encounters. All covariates were calculated at either a physician-day or physician-week level to align with the unit of analysis. We present results separately for each study site to account for potential institutional differences in telemedicine adoption, EHR customization, and workflow, ensuring that findings are relevant across varied healthcare settings.

To confirm the appropriateness of the fixed effects approach, we found that 76.6% of WashU physicians and 100% of UCSF physicians exhibited variation in telemedicine exposure across weeks, indicating that a substantial portion of our sample contributed directly to the within-physician effect estimates.

## Results

### Sample characteristics

In the post-COVID onset period, the WashU sample included 717 attending physicians (319 [44.5%] female) across 11 specialties, and 1 136 858 completed ambulatory visits (Table 1). Together, this includes 85 797 unique physician-days. At WashU, most physician-days were entirely face-to-face (70.6%), followed by ≤50% mixed telemedicine days (24.9%) and entirely telemedicine days (3.3%). The UCSF sample included 585 attending physicians (329 [56.2%] female) and 353 079 completed ambulatory visits, comprising 42 785 physician-days. Among these physician-days, all telemedicine days were most common (31.5%) followed closely by ≤50% mixed telemedicine days (31.4%), and all face-to-face days (27.6%). For full details of sample characteristics in both pre- and post-COVID onset periods, see Table S1—Sample characteristics.

**Table 1.** Sample characteristics.

	WashU (*N* = 717 physicians)	UCSF (*N* = 585 physicians)
Female, *N* (%)	319 (44.5%)	329 (56.2%)
Specialty group, *N* (%)		
Primary care physicians	365 (50.91%)	248 (42.4%)
Medical sub-specialists	289 (40.31%)	287 (49.1%)
Surgical sub-specialists	63 (8.79%)	50 (8.6%)
Clinical specialty, *N* (%)		
Internal medicine	119 (16.0%)	104 (17.8%)
Family medicine	87 (11.7%)	18 (3.1%)
Obstetrics & gynecology	74 (10.0%)	59 (10.1%)
General pediatrics	64 (8.6%)	29 (5.0%)
Dermatology	21 (2.8%)	38 (6.5%)
Neurology	129 (17.3%)	145 (24.8%)
Cardiology	104 (14.0%)	75 (12.8%)
Hematology and oncology	30 (4.0%)	35 (6.0%)
Nephrology	26 (3.5%)	32 (5.5%)
General surgery	24 (3.2%)	14 (2.4%)
Otolaryngology	39 (5.2%)	36 (6.2%)
Completed ambulatory visits, *N*	1 136 858	353 079
Completed E&M visits, *N*	757 557	275 346
Physician-days, *N* (%)		
No telemedicine visits (entirely face-to-face)	60 701 (70.6%)	11 825 (27.6%)
≤50% Telemedicine visits	21 319 (24.9%)	13 429 (31.4%)
>50% Telemedicine visits	910 (1.06%)	4068 (9.5%)
Entirely telemedicine visits	2867 (3.3%)	13 457 (31.5%)
Scheduled patient care hours per day, *m* (SD)	4.6 (2.1)	4.1 (3.1)
Patient encounters per day, *m* (SD)	13.3 (7.6)	8.3 (6.0)

Values shown for post-COVID onset period (March 1, 2021-March 7, 2022) only.

### Unadjusted descriptive statistics

#### Changes in EHR use from pre- to post-COVID onset

**Figure 1.**
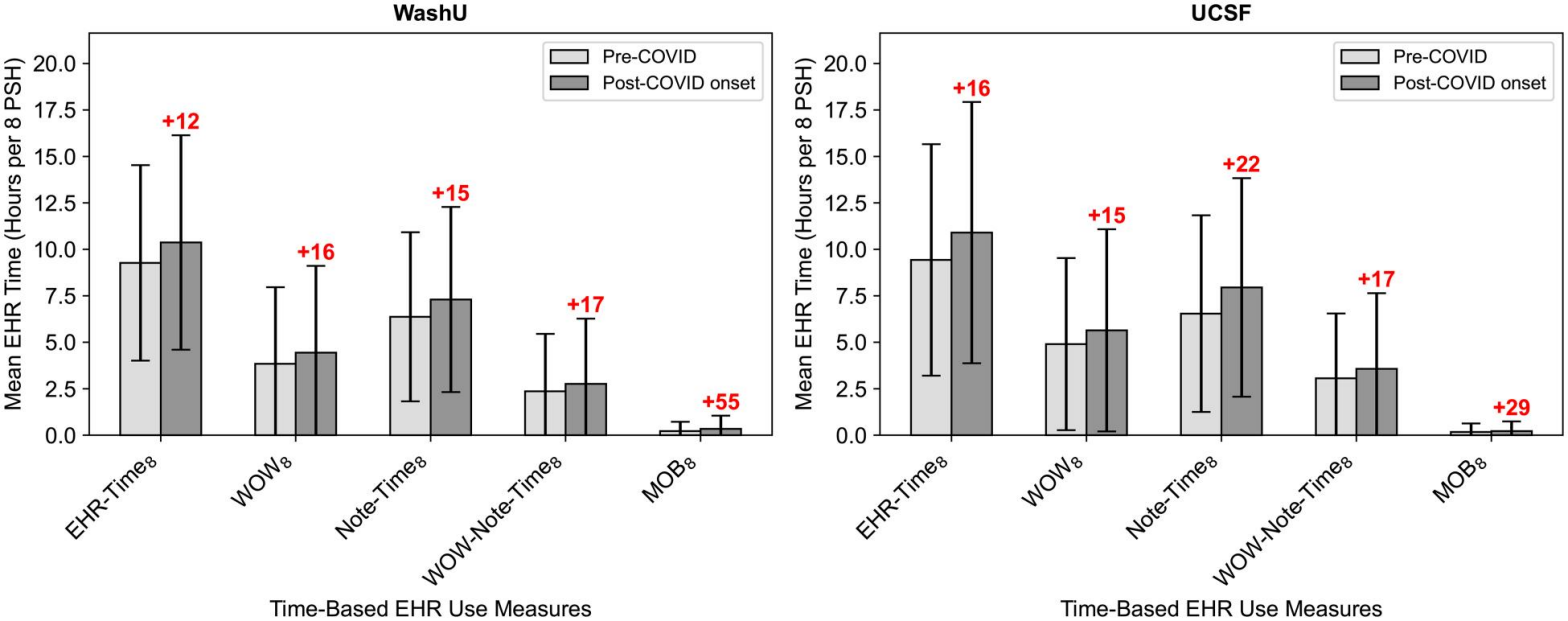
Unadjusted changes in mean EHR time per physician-week pre to post-COVID onset. All time-based measures presented in the figure were normalized per 8 PSHs, and averaged (*m*, SD) at the physician-week level. Each shaded bar represents the mean (*m*), and error bar represents the SD. The colored annotation above each bar corresponding to the post-COVID onset period represents the % difference compared to pre-COVID period, with red denoting a positive difference and blue denoting a negative difference.

In the post-COVID onset period, unadjusted mean EHR time per 8 PSHs increased at both WashU and UCSF (Figure 1). Total EHR time (EHR-Time_8_; 1.1 hours or 12% higher at WashU, 1.8 hours or 16% at UCSF) and documentation time (Note-Time_8_; 0.9 hours or 15% higher at WashU, 0.7 hours or 22% at UCSF) both during and outside of regular working hours were higher in the post-COVID onset period (see Table S2—Changes in physician EHR time pre- vs post-COVID onset periods).

Several discrete EHR tasks saw a post-COVID onset increase, particularly information seeking activities: chart review actions, rose from 158.4 to 190.4 per physician-day at WashU, and 85.6 to 104.5 at UCSF (see Table S3—Changes in counts of EHR tasks pre to post-COVID onset). We observed the largest change in clinical communication activities, with patient-messages viewed changed from 2.3 to 5.1, and patient-messages sent changed from 1.3 to 2.7 at WashU. Changes at UCSF were 5.0 to 7.3 and 3.3 to 4.6.

#### Encounter modality and EHR use measures (post-COVID onset period)

**Figure 2.**
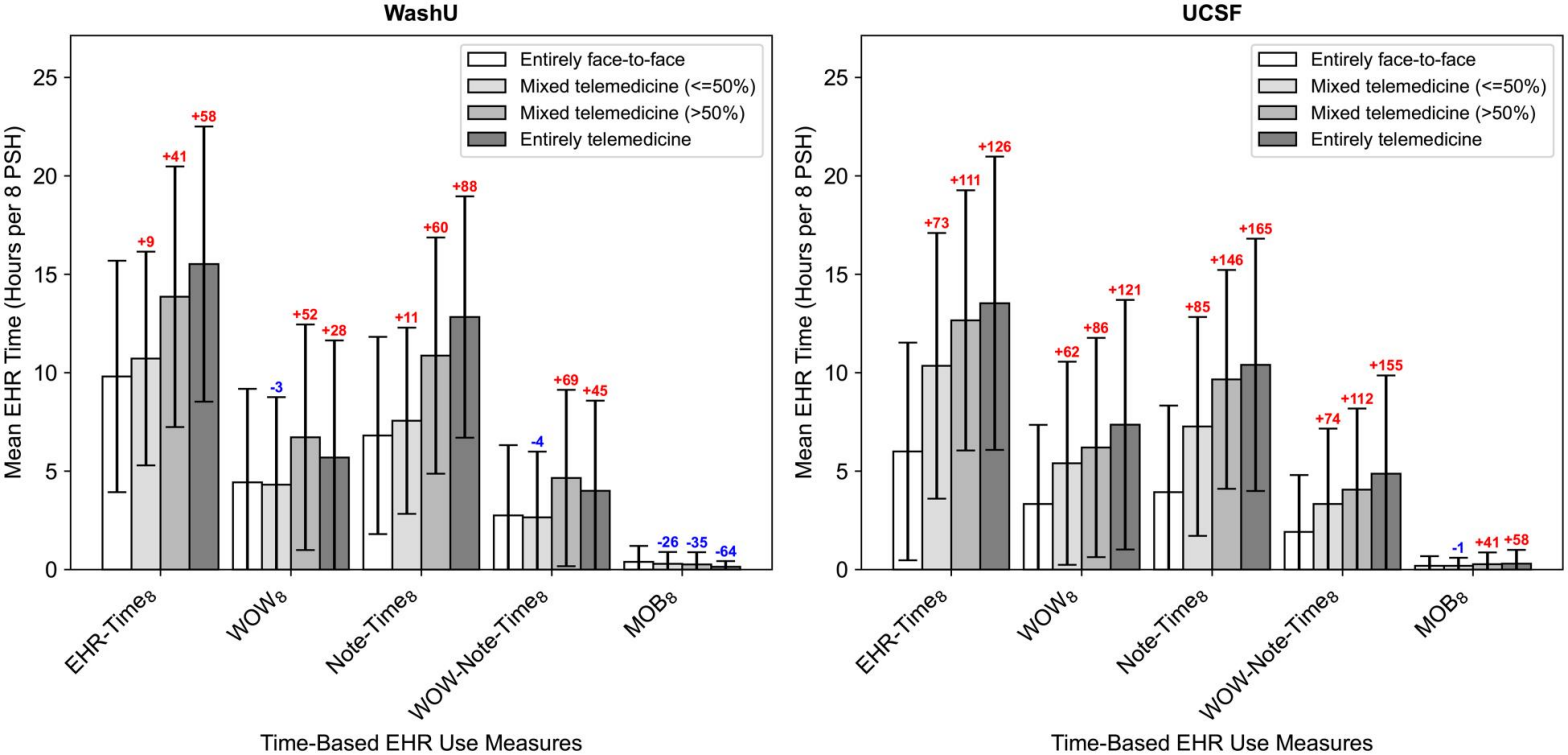
Unadjusted changes in mean EHR time per physician-week by visit modality mix (ie, telemedicine intensity) in the post-COVID onset period. All time-based measures presented in the figure were normalized per 8 PSHs and averaged per physician-week (*m*, SD). Each shaded bar represents the mean (*m*), and error bar represents the SD. The colored annotation above each bar corresponding to each telemedicine encounter modality category represents the % difference compared to all face-to-face encounter modality category, with red denoting a positive difference and blue denoting a negative difference.

In the post-COVID onset period, unadjusted mean EHR time varied significantly across visit modality categories. Compared to WashU, UCSF exhibited a more monotonic relationship, with all EHR time-based measures increasing as the proportion of telemedicine visits rose—except for time spent on mobile devices (Figure 2). At both WashU and UCSF, total EHR time (EHR-Time_8_) and documentation time (Note-Time_8_) were greater in physician-weeks with higher telemedicine intensity compared to entirely face-to-face weeks (see Table S4—Changes in physician EHR time across telemedicine encounter modalities). At WashU, fewer EHR hours on mobile devices (MOB_8_) were observed in weeks with higher telemedicine intensity compared to entirely face-to-face weeks, whereas at UCSF, longer MOB_8_ hours were observed in >50% mixed telemedicine weeks. WashU physicians’ EHR use outside of scheduled hours (eg, WOW_8_ and WOW-Note-Time_8_) exhibited mixed patterns: ≤50% mixed telemedicine weeks had fewer WOW_8_ and WOW-Note-Time_8_ hours than entirely face-to-face weeks, while >50% mixed telemedicine weeks showed longer hours. In contrast, at UCSF, WOW_8_ and WOW-Note-Time_8_ hours consistently increased in physician-weeks with higher telemedicine intensity.

Similarly, unadjusted frequency-based measures of EHR use varied significantly across visit modality categories (see Table S5—Changes in counts of EHR tasks across telemedicine encounter modalities). At WashU, while ≤50% mixed telemedicine physician-days showed more frequent EHR activity compared to entirely face-to-face days, and overall decreasing trend in EHR use was observed across all frequency-based measures as the proportion of scheduled telemedicine visits increased. At UCSF, the relationship between proportion of telemedicine encounters and EHR actions was often non-linear. For example, mixed telemedicine days had a higher number of outside records views than entirely telemedicine days.

### Adjusted multivariable regression analysis of the association between telemedicine modality and EHR use

**Figure 3.**
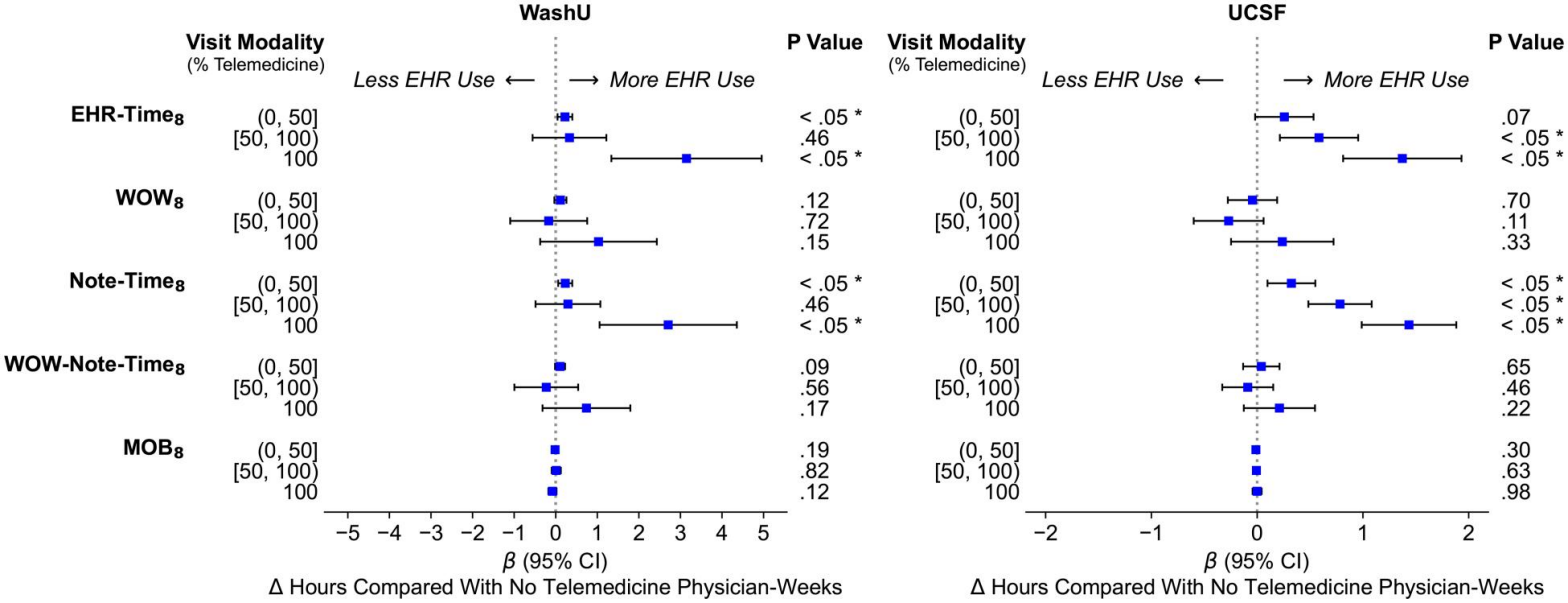
Adjusted changes in mean EHR time per physician-week by visit modality mix (ie, telemedicine intensity) in post-COVID onset period (*: *P* < .05). All time-based measures presented in the figure were normalized per 8 PSHs.

Entirely telemedicine physician-weeks was associated with increased time spent on EHR (EHR-Time_8_) and documentation (Note-Time_8_) during PSHs (Figure 3). At WashU, physicians spent 3.1 total additional hours on EHR per 8 PSHs (*P* < .001; 95% CI, 1.3-5.0) and 2.7 total additional hours of documentation per 8 PSHs (*P* = .002; 95% CI, 1.1-4.4) in entirely telemedicine weeks compared with entirely face-to-face weeks. UCSF physicians working in entirely telemedicine weeks spent an additional 1.4 hours on the EHR per 8 PSHs (*P* < .001; 95% CI, 0.8-1.9) and 1.4 additional hours of documentation work per 8 PSHs (*P* < .001; 95% CI, 1.0-1.9) compared with entirely face-to-face weeks. At both institutions, mixed modality weeks also showed increased EHR time, although with mixed statistical significance.

**Figure 4.**
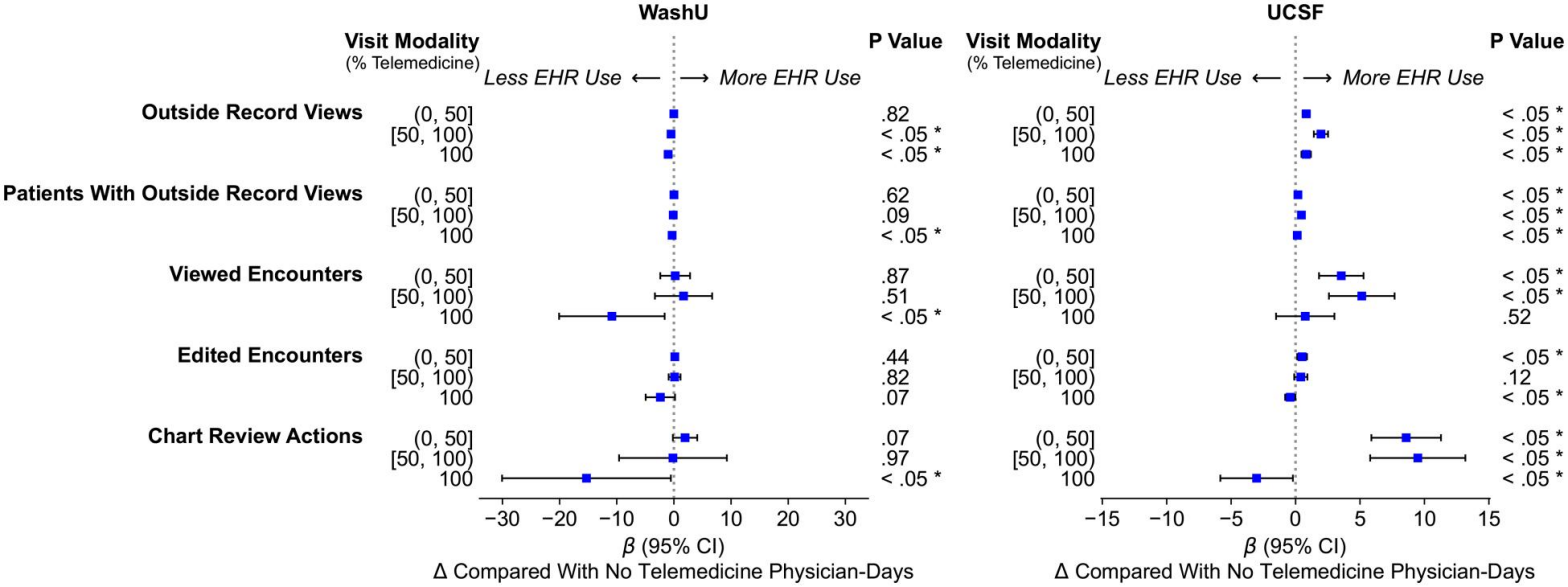
Adjusted changes in the frequency of EHR activity related to *information seeking* per physician-day by visit modality mix (ie, telemedicine intensity) in post-COVID onset period (*: *P* < .05).

We saw a mixed pattern of change in *information-seeking* behaviors across different modality categories across institutions (Figure 4). At WashU, information-seeking behaviors decreased with telemedicine use. Physicians in entirely telemedicine days performed one fewer outside record views per day (*P* < .001; 95% CI, −1.5 to −0.6) compared to entirely face-to-face days, whereas those in >50% mixed telemedicine days showed a small decrease, with 0.5 fewer outside record views per day (*P* = .036; 95% CI, −1.0 to −0.0). In contrast, at UCSF, information-seeking behaviors increased significantly with telemedicine use. Physicians in entirely telemedicine days performed an additional 0.8 outside record views per day (*P* < .001; 95% CI, 0.5-1.2), while those in >50% mixed telemedicine days showed an even larger increase, with 2.0 additional outside record views per day (*P* < .001; 95% CI, 1.4-2.5). For ≤50% mixed telemedicine days, the increase was smaller but still significant at 0.8 actions (*P* < .001; 95%CI, 0.5-1.1).

**Figure 5.**
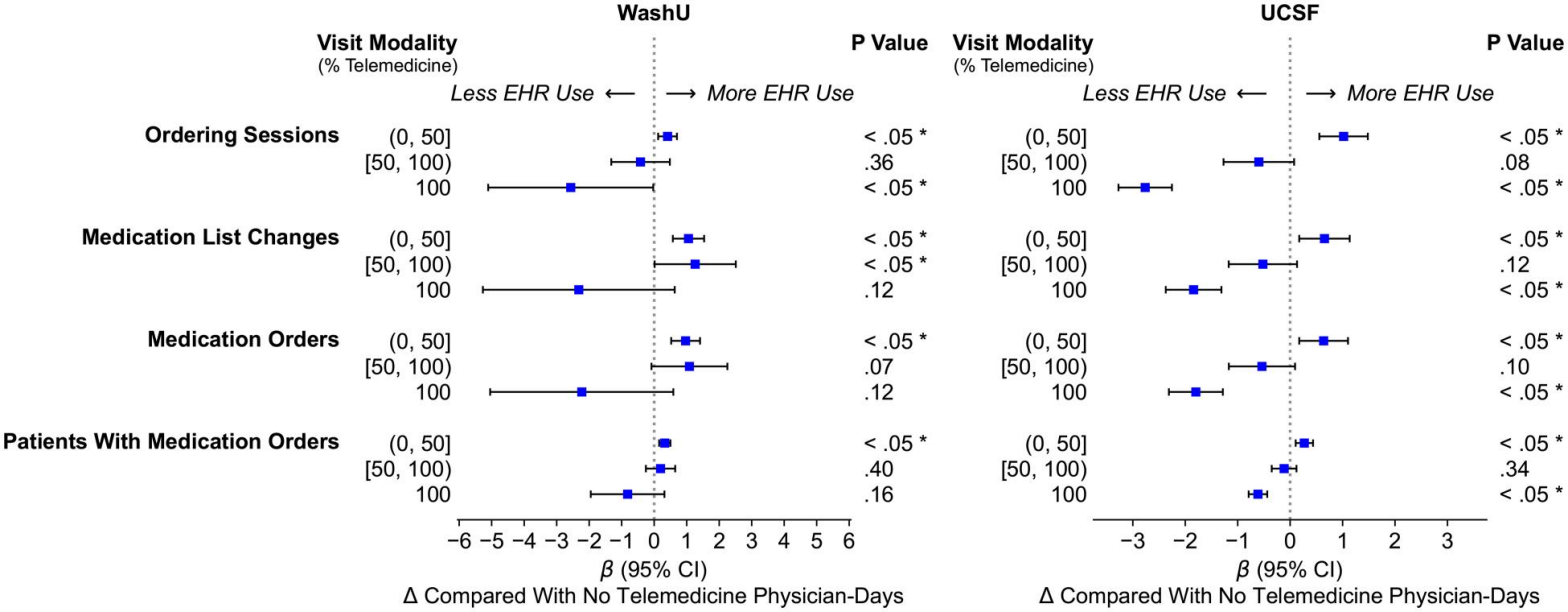
Adjusted changes in the frequency of EHR activity related to *ordering intensity* per physician-day by visit modality mix (ie, telemedicine intensity) in post-COVID onset period (*: *P* < .05).

For *ordering intensity*, entirely telemedicine days were associated with decreased ordering sessions at WashU, with a 2.6 fewer ordering sessions per day (*P* = .047; 95% CI, −5.1 to −0.0) compared to face-to-face days (Figure 5). The >50% mixed telemedicine days also saw a decrease of 0.4 ordering sessions per day (*P* > .05; 95% CI, −1.3 to 0.5), while ≤50% mixed telemedicine days showed a slight increase of 0.4 more ordering sessions per day (*P* = .005; 95% CI, 0.1-0.7). Similarly, at UCSF, entirely telemedicine days were associated with fewer ordering sessions, with a decrease of 2.8 orders per day (*P* < .001; 95% CI, −3.3 to −2.3) compared to face-to-face days. The >50% mixed telemedicine days also saw a decrease of 0.6 ordering sessions per day (*P* > .05; 95% CI, −1.3 to 0.1), while ≤50% mixed telemedicine days showed a slight increase of one ordering sessions per day (*P* < .001; 95% CI, 0.6-1.5).

**Figure 6.**
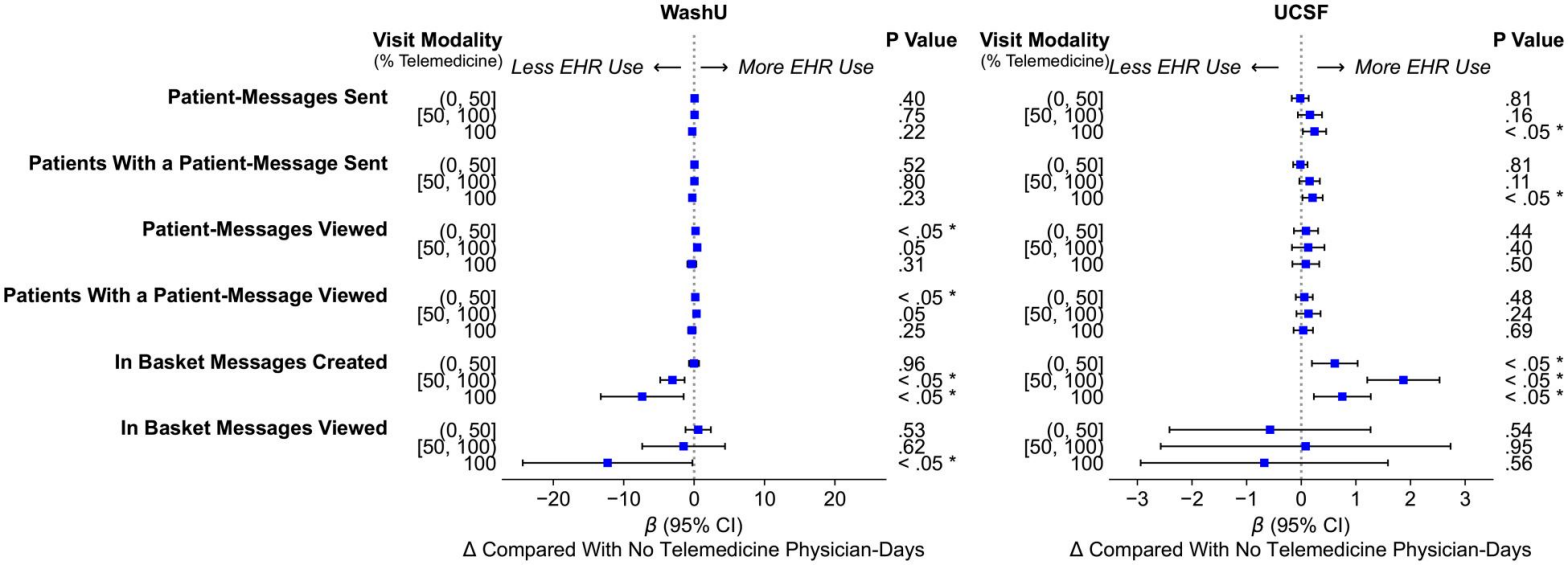
Adjusted changes in the frequency of EHR activity related to *clinical communication* per physician-day by visit modality mix (ie, telemedicine intensity) in post-COVID onset period (*: *P* < .05).

*Clinical communication* frequency metrics showed mixed results across institutions (Figure 6). At WashU, physicians in entirely telemedicine days showed 0.4 more patient-messages viewed per day (*P* > .05; 95% CI, −0.0 to 0.9). In contrast, those in ≤50% mixed telemedicine days showed 0.2 more patient-messages viewed per day (*P* = .014; 95% CI, 0.0-0.4). However, entirely telemedicine days generally saw a statistically nonsignificant decrease in all clinical communication metrics. On the other hand, at UCSF, we generally saw an increase in most clinical communication metrics across all telemedicine intensity categories, despite with mixed statistical significance. Physicians in entirely telemedicine days showed 0.1 more patient-messages viewed per day (*P* > .05; 95% CI, −0.2 to 0.3), while those in >50% mixed telemedicine days showed 0.1 more patient-messages viewed per day (*P* > .05; 95% CI, −0.2 to 0.4). For full details of adjusted statistical analysis results, see Table S6—Changes in physician EHR time pre- vs post-COVID onset periods.

## Discussion

This multi-site study of physician ambulatory EHR use assessed the impact of telemedicine on time spent in the EHR and discrete EHR tasks. We observed changes in EHR use between the pre- and post-COVID onset periods, including increases in total EHR time and documentation time. Within the post-COVID, at both WashU and UCSF, we found that an increased proportion of telemedicine visits was associated with total EHR time and time spent on documentation (both overall and outside of regular working hours) and fewer activities related to ordering intensity. Meanwhile, we also found mixed patterns regarding the use of specific EHR functions across 2 institutions, including those related to information seeking activity and clinical communication. Our results highlight how telemedicine use was associated with shifts in not only how much time was spent on EHR-based activities, but also the types of EHR activities that were performed (eg, information seeking, ordering, clinical communication). These results underscore the need for health systems and EHR vendors to optimize EHR tools, support structures (eg, team composition and tasks), and clinical workflows to accommodate the unique needs of telemedicine care delivery.

The relationship between the proportion of telemedicine encounters (compared to total encounters) and EHR use were not always monotonic, especially across the frequency-based measures representing discrete EHR tasks. This could potentially be related to a number of factors: it is possible that there are “switching costs” to delivering both in-person and telemedicine care on the same day, such that physicians practice differently on mixed-modality days. Although we did not directly measure switching costs, we hypothesize that it is a key mechanism underlying the observed differences in EHR engagement across telemedicine intensities. Future work could empirically assess these costs by tracking workflow disruptions or time fragmentation across visit modalities. It may also be the case that scheduling is conducted differently for entirely telemedicine days (vs mixed-modality days) in a way that our observable covariates are unable to adjust for. However, the consistently greater EHR time for telemedicine days highlight the need for increased support for physicians delivering care virtually to balance patient access to telemedicine care with physician well-being, given ongoing concerns over EHR-induced physician burnout.^18^

Both institutions saw a decrease in order-related actions on telemedicine days, suggesting that physicians’ clinical decision-making processes may be constrained when delivering care via telemedicine. This highlights the challenges of virtual care, where lack of direct patient contact may limit physicians’ ability to initiate or adjust treatment plans as they would in face-to-face encounters. However, it is also possible that telemedicine visits are intentionally structured to be less complex, focusing on conditions that require fewer diagnostic tests or treatment adjustments. This suggests that the observed reduction in order-related actions may reflect not just limitations of telemedicine but also differences in case complexity or clinical focus. While recent research has shown that telemedicine can reduce inefficiencies in care delivery, our findings highlight a different operational impact—increased physician EHR tasks. Ganguli et al found modest reductions in low-value testing and spending with telemedicine adoption, despite a small rise in visit volume.^19^ In contrast, we observed that telemedicine encounters were consistently linked to greater EHR engagement, particularly for documentation time. This suggests a possible trade-off: while telemedicine may reduce ordering and unnecessary testing, it may simultaneously shift administrative burdens to physicians. Addressing this workload shift will be essential for ensuring sustainable telemedicine integration.

Interestingly, we observed unexpected differences in EHR use patterns associated with telemedicine across the 2 study sites, despite both institutions being large academic medical centers with shared goals of expanding telemedicine access, especially at the onset of the COVID pandemic. At UCSF, as the proportion of telemedicine visits increased, physicians engaged more frequently in information seeking and clinical communication activities, whereas at WashU, the opposite trend was observed. These differences could reflect prior institutional experience with telemedicine, degree of integration between telehealth platforms and the EHR, or regional differences in care demands. UCSF’s location within a dense and competitive healthcare market may suggest that telemedicine could increase the value of outside records and patient messaging due to greater inter-organizational care fragmentation, but only in markets with patients receiving care at multiple institutions.

Another potential explanation for differences in communication-related EHR activity is the physical co-location of physicians and clinic staff. When both parties are remote, physicians may rely more heavily on asynchronous messaging tools, whereas on-site co-location may facilitate informal, real-time coordination. Although our audit log data do not capture workstation locations or staff presence, future research could incorporate such information to better understand how co-location dynamics shape messaging patterns during virtual care.

Our findings highlight several areas where healthcare systems may need to adapt to support sustainable telemedicine workflows, such as allocating additional resources in the form of team-based support and AI-assisted tools, to help physicians manage EHR time demands—particularly when such demands reflect increased multitasking or fragmented workflows. Operational strategies should focus on optimizing hybrid care delivery, where physicians balance telemedicine with face-to-face visits, and restructuring teams to better address the demands of virtual care. These findings also point to a potential need for formal training programs that prepare clinicians to manage telemedicine-specific workflows. Such training could address documentation during telemedicine visits, effective EHR use during virtual care, and strategies to structure hybrid care models to minimize workflow fragmentation and support clinician focus. At the same time, we acknowledge that increased EHR time in telemedicine does not necessarily indicate increased burden. Differences in EHR engagement may reflect variations in multitasking or uninterrupted documentation workflows, rather than added strain. Future research using survey-based or qualitative approaches could help assess how clinicians perceive EHR workload across care modalities.

To ensure physicians are adequately supported, health systems, EHR vendors, and clinicians should assess how EHR use evolves—particularly in response to the rapid expansion of telemedicine—to balance patient access, physician well-being, and EHR burden. Policymakers may wish to explore alternative reimbursement levels for virtual care given the additional time cost of telemedicine.

### Limitations

These results should be interpreted with a number of limitations in mind. First, our data is observational, and while we use fixed effects regression models to adjust for both observed and unobserved confounders, our findings do not establish causality. Further, we are not able to observe or adjust for the specific clinical issue(s) addressed in each encounter, which limits interpretation of how to adapt the EHR for telemedicine workflows. Because our models estimate within-physician associations, only physicians with variation in telemedicine exposure contributed to the telemedicine effect estimates; we did not quantify this subset, but this may modestly reduce the effective sample size relative to the full analytic cohort. Second, while our study is one of the first to span multiple health systems, both sites are academic centers, and the impact of telemedicine may differ in other healthcare settings, such as federally qualified health centers or safety-net clinics. Moreover, we analyzed the 2 institutions separately rather than pooling the data, as the systems differ significantly in their geographic regions, patient populations, and practice patterns. Given these differences, a combined analysis could obscure meaningful variations in how telemedicine affects EHR use, reinforcing the need for site-specific insights. Telemedicine platform integration into the EHR also differed across sites—most visits at UCSF were non-integrated, whereas those at WashU were largely integrated into the EHR. However, both sites showed similar associations between telemedicine use and documentation time, suggesting integration (specifically whether time spent on video is counted as EHR time) is unlikely to explain our findings. Finally, our data is aggregated to the day- and week-level, rather than the encounter level, which limits our ability to distinguish changes in EHR use driven by telemedicine from those influenced by scheduling practices or patient selection into virtual care. Future work with more granular data could help disentangle these effects and provide a clearer understanding of how telemedicine influences EHR engagement.

We used the established “per 8 scheduled patient hours” metric to normalize EHR time across different clinical volumes.^17^ Although appointment durations may vary by modality, our models adjusted for scheduled visit length and complexity, and “per visit” denominators could obscure the broader structural effects of telemedicine visit volume and appointment length. Similarly, although interface changes (eg, Epic Storyboard) may influence EHR use patterns, our use of week-based fixed effects helps account for secular system-wide changes. Finally, some increases in EHR time during telemedicine may reflect more active or continuous engagement rather than increased burden, and not all audit log-measured time is necessarily experienced as disruptive.

Additionally, while we separately analyzed medication orders, our audit log data do not differentiate diagnostic orders (eg, labs, imaging) from other procedural or therapeutic orders. This single event metric aggregated multiple ordering types. Future studies using more granular order-level data may be able to better distinguish how specific types of clinical decision-making are influenced by care modality.

## Conclusion

In this multisite study of the relationship between telemedicine and EHR use, we found that greater telemedicine intensity in ambulatory care was associated with greater EHR time and fewer orders, but mixed results across EHR tasks related to information seeking and clinical communication. There was variation between 2 large academic medical centers that likely reflects differences in when and how telemedicine is used as well as broader factors such as their geographic regions, patient populations, and practice patterns.

## Supplementary Material

ocaf122_Supplementary_Data

## Data Availability

The data underlying this study cannot be shared publicly because it includes patient-identifying information that cannot be reasonably removed without compromising the quality of the shareable data.

## References

[1] Smith AC , Thomas E, Snoswell CL, et al Telehealth for global emergencies: implications for coronavirus disease 2019 (COVID-19). J Telemed Telecare. 2020;26:309-313. 32196391 10.1177/1357633X20916567 PMC7140977

[2] Bakken S. Telehealth: Simply a Pandemic Response or Here to Stay?. Oxford University Press; 2020:989-990. 10.1093/jamia/ocaa132 PMC7647362 32692845

[3] Patel PD , Cobb J, Wright D, et al Rapid development of telehealth capabilities within pediatric patient portal infrastructure for COVID-19 care: barriers, solutions, results. J Am Med Inform Assoc. 2020;27:1116-1120. 32302395 10.1093/jamia/ocaa065 PMC7188108

[4] Wosik J , Fudim M, Cameron B, et al Telehealth transformation: COVID-19 and the rise of virtual care. J Am Med Inform Assoc. 2020;27:957-962. 32311034 10.1093/jamia/ocaa067 PMC7188147

[5] Affairs OoPaI. VA Video Connect visits increase 1000% during COVID-19 pandemic. 2020. Accessed June 8, 2025. https://connectedcare.va.gov/whats-new/technology/va-video-connect-visits-increase-1000-during-covid-19-pandemic

[6] Office of the Assistant Secretary for Planning and Evaluation. ASPE Issue Brief: Medicare Beneficiary Use of Telehealth Visits: Early Data From the Start of the COVID-19 Pandemic. 2020. Accessed June 8, 2025. https://aspe.hhs.gov/reports/medicare-beneficiary-use-telehealth-visits-early-data-start-covid-19-pandemic

[7] Bestsennyy O , Gilbert G, Harris A, et al Telehealth: a quarter-trillion-dollar post-COVID-19 reality. McKinsey & Company. 2021;9. Accessed June 8, 2025. https://www.mckinsey.com/industries/healthcare/our-insights/telehealth-a-quarter-trillion-dollar-post-covid-19-reality

[8] Bashshur R , Doarn CR, Frenk JM, et al Telemedicine and the COVID-19 pandemic, lessons for the future. Telemed J E Health. 2020;26:571-573. 32275485 10.1089/tmj.2020.29040.rb

[9] Asan O. Providers’ perceived facilitators and barriers to EHR screen sharing in outpatient settings. Appl Ergon. 2017;58:301-307. 27633226 10.1016/j.apergo.2016.07.005

[10] Montague E , Asan O. Dynamic modeling of patient and physician eye gaze to understand the effects of electronic health records on doctor–patient communication and attention. Int J Med Inform. 2014;83:225-234. 24380671 10.1016/j.ijmedinf.2013.11.003 PMC4046907

[11] Asan O , Montague E. Using video-based observation research methods in primary care health encounters to evaluate complex interactions. Inform Primary Care. 2014;21:161-170. 25479346 10.14236/jhi.v21i4.72 PMC4350928

[12] Gopnik A. The New Theatrics of Remote Therapy. *The New Yorker*. 2020. Accessed June 8, 2025. https://www.newyorker.com/magazine/2020/06/01/the-new-theatrics-of-remote-therapy

[13] Agrawal S , Gandhi T. Telehealth should be expanded—if it can address today’s health care challenges. *Health Affairs Forefront*. 2020. Accessed June 8, 2025. https://www.healthaffairs.org/content/forefront/telehealth-should-expanded-if-can-address-today-s-health-care-challenges

[14] Shaarani I , Taleb R, Antoun J. Effect of computer use on physician-patient communication using a validated instrument: patient perspective. Int J Med Inform. 2017;108:152-157. 29132621 10.1016/j.ijmedinf.2017.10.007

[15] Holmgren AJ , Thombley R, Sinsky CA, et al Changes in physician electronic health record use with the expansion of telemedicine. JAMA Intern Med. 2023;183:1357-1365. 37902737 10.1001/jamainternmed.2023.5738 PMC10616769

[16] Adler-Milstein J , Adelman JS, Tai-Seale M, et al EHR audit logs: a new goldmine for health services research? J Biomed Inform. 2020;101:103343. 10.1016/j.jbi.2019.103343 31821887

[17] Sinsky CA , Rule A, Cohen G, et al Metrics for assessing physician activity using electronic health record log data. J Am Med Inform Assoc. 2020;27:639-643. 32027360 10.1093/jamia/ocz223 PMC7075531

[18] Adler-Milstein J , Zhao W, Willard-Grace R, et al Electronic health records and burnout: time spent on the electronic health record after hours and message volume associated with exhaustion but not with cynicism among primary care clinicians. J Am Med Inform Assoc. 2020;27:531-538. 10.1093/jamia/ocz220 32016375 PMC7647261

[19] Ganguli I , Lim C, Daley N, et al Telemedicine adoption and low-value care use and spending among fee-for-service medicare beneficiaries. JAMA Intern Med. 2025;185:440-449. 39992684 10.1001/jamainternmed.2024.8354 PMC11851298

