# Supplementary material for: Differences in physician electronic health record use by telemedicine intensity: evidence from 2 academic medical centers
Source: J Am Med Inform Assoc. 2025 Jul 11;32(9):1462–70. doi: 10.1093/jamia/ocaf122 (PMC12361853; doi:10.1093/jamia/ocaf122)
Supplement: ocaf122_Supplementary_Data [file ocaf122_supplementary_data.docx]

Differences in Physician EHR Use by Telemedicine Intensity: Evidence from Two Academic Medical Centers

Seunghwan Kim, MS,^1,3,§^ Robert Thombley^2^, Elise Eiden, MS,^4^ Sunny S. Lou, MD, PhD,^3,4^ Julia Adler-Milstein, PhD,^2^ Thomas Kannampallil, PhD,^3,4^ and A Jay Holmgren, PhD MHI^2^

^1^Roy and Diana Vagelos Division of Biology and Biomedical Sciences, Washington University School of Medicine in St. Louis, Saint Louis, USA

^2^Division of Clinical Informatics and Digital Transformation, Department of Medicine, University of California, San Francisco, San Francisco, CA, USA

^3^Institute for Informatics, Data Science, and Biostatistics (I2DB), Washington University School of Medicine in St. Louis, Saint Louis, USA

^4^Department of Anesthesiology, Washington University School of Medicine in St. Louis, Saint Louis, USA

^§^*Corresponding author*:

Seunghwan Kim, MS

4939 Children's Place

St Louis, Missouri 63110

[seunghwan.kim@wustl.edu](mailto:seunghwan.kim@wustl.edu)

(+1)314.273.7801

#

# Supplementary Methods

Computer code is available at <https://github.com/thomas-k-wustl/telemedicine_EHR_use>. Data processing and statistical analysis were performed using the statsmodels package in Python 3.11.4 (Python Software Foundation, Wilmington, DE, USA) and the fixest library in R 4.1.1 (The R Foundation for Statistical Computing, Vienna, Austria). The analytic code used in this study can be made available upon reasonable request, contingent upon appropriate institutional agreements. Access may be subject to restrictions to protect proprietary information contained within the EHR audit logs and associated system configurations.

## Frequency-based EHR usage measures

### Information seeking activities

When measuring EHR activities related to viewing outside records of patients (i.e., Care Everywhere in Epic), we included all actions related to outside record-viewing activity recorded in the audit logs.

When measuring EHR activities related to interacting with internal patient charts, we used unique identifiers of patient encounters, since patient chart can be defined as any activity related to a specific patient encounter. For measuring EHR activities related to documented internal patient charts, we only included encounter identifiers for any action related to modification.

### Ordering intensity

To measure total count of overall order sessions, we counted distinct occurrences of actions related to creation of an order list. Furthermore, to calculate the number of medication modification activities, we used a table that tracks all order status-change events to identify all sign, delete or modify events for medication-specific orders that were performed by each physician on a given day. We calculated the cumulative total of all events performed on that user-day, the total number of impacted orders on that user-day, and the total number of impacted patients on that user-day.

### Communication

To measure patient EHR portal messaging activities, we counted distinct occurrences of actions related to sending of a patient message, and those related to viewing of an inbox message. In order to select only the patient portal messages among inbox messages, we filtered the messages to only include those with an appropriate message type code. To measure broader electronic inbox activities, we counted distinct occurrences of actions related to message creation and message viewing.

An overview of frequency-based measures across the three categories is shown in Table A.

**Table A.** Breakdown of frequency-based EHR usage measures by category.

| **EHR Use Category** | **Sub-category** | **Constructed Measure** |
| --- | --- | --- |
| Information Seeking | Outside Records Viewed | Outside record views |
|  |  | Patients with outside record views |
|  | Patient Chart Review | Viewed encounters |
|  |  | Edited encounters |
|  |  | Chart review actions |
| Ordering Intensity | Order Sessions | Ordering sessions |
|  | Medications Modified | Medication list changes |
|  |  | Medication orders |
|  |  | Patients with medication orders |
| Communication | MyChart Patient Messages Sent / Received | Patient-message sent |
|  |  | Patients with a patient-message sent |
|  |  | Patient-messages viewed |
|  |  | Patients with a patient-message viewed |
|  | In Basket Messages | In Basket messages created |
|  |  | In Basket messages viewed |

## Statistical analysis

### Descriptive statistics

We calculated sample characteristics for the study sample over two 1-year periods, before and during the COVID-19 pandemic (pre-COVID and post-COVID, accordingly). We measured volume of telemedicine use in ambulatory clinical work stratified by encounter modality categories (e.g., fully telemedicine, mixed telemedicine and face-to-face, and fully face-to-face visits), including the total counts of workdays, average counts of evaluation and management (E/M) encounters per workday, average E/M work relative value units (RVUs) per encounter per workday, and average proportion of level 4/5 E/M encounters per workday (which may indicate patient complexity). All measures related to telemedicine volume were calculated based on scheduled and completed ambulatory encounters. We calculated demographic characteristics such as the count of attending physicians in the sample by specialty, and by specialty group (i.e., primary specialty, medical specialty, surgical specialty). We also calculated the general volume of work, including average scheduled patient care hours per workday, average count of scheduled patient encounters per workday, total completed encounter volume, and total completed E/M encounters.

We calculated descriptive statistics of unadjusted EHR usage variables including time- and count-based EHR usage measures. Time-based measures included weekly EHR-Time_8_, WOW_8_, Note-Time_8_, WOW-Note-Time_8_, and MOB_8_ as defined in the main text. Frequency-based measures were calculated at the physician-day level, and measured EHR activities related to three usage categories: information seeking behaviors, ordering intensity, and communication.

We calculated the time- and frequency-based EHR usage measures for the study sample over the two 1-year periods (i.e., pre- and post-COVID periods) and used 2-sided univariate Mann-Whitney U tests to test for differences in the distribution of each unadjusted EHR usage variable.

Similarly, we calculated all EHR usage measures again just on the post-COVID period, with each provider-day stratified into one of the four telemedicine encounter modality categories defined as: entirely face-to-face, ≤50% mixed telemedicine, >50% mixed telemedicine, and entirely telemedicine. We used 2-sided univariate Mann-Whitney U tests to test for differences in the distribution of each unadjusted EHR usage variable between encounter modalities, with using entirely face-to-face visits as the reference (e.g., entirely face-to-face vs. telemedicine modality category).

# Supplementary Results

**Table S1**. Sample characteristics. Values shown for both pre- and post-COVID onset periods.

|  | **WashU** | | **UCSF** | |
| --- | --- | --- | --- | --- |
| **Study Period** | **Pre-COVID** | **Post-COVID** | **Pre-COVID** | **Post-COVID** |
| Physician, N | 635 | 717 | 553 | 585 |
| Female, N (%) | 279 (43.9%) | 319 (44.5%) | 295 (53.3%) | 329 (56.2%) |
| Specialty Group, N (%) |  |  |  |  |
| Primary Care Physicians | 313 (49.3%) | 365 (50.91%) | 233 (42.1%) | 248 (42.4%) |
| Medical Sub-Specialists | 264 (41.57%) | 289 (40.31%) | 274 (49.6%) | 287 (49.1%) |
| Surgical Sub-Specialists | 58 (9.1%) | 63 (8.79%) | 46 (8.3%) | 50 (8.6%) |
| Clinical Specialty, N (%) |  |  |  |  |
| Internal Medicine | 96 (14.4%) | 119 (16.0%) | 100 (18.1%) | 104 (17.8%) |
| Family Medicine | 71 (10.6%) | 87 (11.7%) | 18 (3.3%) | 18 (3.1%) |
| Obstetrics & Gynecology | 66 (9.9%) | 74 (10.0%) | 52 (9.4%) | 59 (10.1%) |
| General Pediatrics | 56 (8.4%) | 64 (8.6%) | 24 (4.3%) | 29 (5.0%) |
| Dermatology | 24 (3.6%) | 21 (2.8%) | 39 (7.1%) | 38 (6.5%) |
| Neurology | 119 (17.8%) | 129 (17.3%) | 137 (24.8%) | 145 (24.8%) |
| Cardiology | 93 (13.9%) | 104 (14.0%) | 74 (13.4%) | 75 (12.8%) |
| Hematology & Oncology | 29 (4.3%) | 30 (4.0%) | 35 (6.3%) | 35 (6.0%) |
| Nephrology | 23 (3.4%) | 26 (3.5%) | 28 (5.1%) | 32 (5.5%) |
| General Surgery | 22 (3.3%) | 24 (3.2%) | 14 (2.5%) | 14 (2.4%) |
| Otolaryngology | 36 (5.4%) | 39 (5.2%) | 32 (5.8%) | 36 (6.2%) |
| Completed Ambulatory Visits, N | 1,032,509 | 1,136,858 | 334,251 | 353,079 |
| Completed E&M Visits, N | 697,085 | 757,557 | 242,996 | 275,346 |
| Physician-Days, N (%) |  |  |  |  |
| No Telemedicine Visits (Entirely Face-to-Face) | 76,329 (100%) | 60,701 (70.6%) | 34,118 (86.8%) | 11,825 (27.6%) |
| ≤50% Telemedicine Visits | 12 (0.0%) | 21,319 (24.9%) | 4,225 (10.8%) | 13,429 (31.4%) |
| >50% Telemedicine Visits | 0 (0.0%) | 910 (1.06%) | 233 (0.6%) | 4,068 (9.5%) |
| Entirely Telemedicine Visits | 0 (0.0%) | 2,867 (3.3%) | 724 (1.8%) | 13,457 (31.5%) |
| Scheduled Patient Care Hours Per Day, *m (SD)* | 4.5 (2.1) | 4.6 (2.1) | 4.1 (3.1) | 4.1 (3.1) |
| Patient Encounters per Day, *m (SD)* | 13.5 (7.7) | 13.3 (7.6) | 8.5 (6.2) | 8.3 (6.0) |

**Table S2**. Changes in physician EHR time pre- versus post-COVID onset periods. Values shown are average EHR time (*m*, SD) per physician-week, normalized per 8 patient scheduled hours (PSHs).

|  | **WashU** | | **UCSF** | |
| --- | --- | --- | --- | --- |
| **Time-based Measures (per Provider-Week)** | **Pre-COVID, *m* (SD)** | **Post-COVID Onset, *m* (SD)** | **Pre-COVID, *m* (SD)** | **Post-COVID Onset, *m* (SD)** |
| EHR-Time_8_ | 9.3 (5.3) | 10.4 (5.8) | 16.7 (9.7) | 18.5 (11.1) |
| WOW_8_ | 3.8 (4.1) | 4.4 (4.7) | 9.4 (6.2) | 10.9 (7.0) |
| Note-Time_8_ | 6.4 (4.6) | 7.3 (5.0) | 4.9 (4.6) | 5.6 (5.4) |
| WOW-Note-Time_8_ | 2.4 (3.1) | 2.8 (3.5) | 6.5 (5.3) | 8.0 (5.9) |
| MOB_8_ | 0.2 (0.5) | 0.3 (0.7) | 3.1 (3.5) | 3.6 (4.1) |

**Table S3**. Changes in counts of EHR tasks pre to post-COVID onset. Values shown are average counts of EHR activity (*m*, SD) per physician-day.

|  | **WashU** | | **UCSF** | |
| --- | --- | --- | --- | --- |
| **Frequency-based Measures (per Provider-Day)** | **Pre-COVID, *m* (SD)** | **Post-COVID Onset, *m* (SD)** | **Pre-COVID, *m* (SD)** | **Post-COVID Onset, *m* (SD)** |
| **Information Seeking** | | | | |
| *Outside Records Viewing* | | | | |
| Outside record views | 3.5 (6.3) | 4.4 (7.5) | 5.8 (9.9) | 6.9 (11.0) |
| Patients with outside record views | 1.5 (2.2) | 1.9 (2.5) | 1.8 (2.1) | 2.0 (2.2) |
| *Patient Chart Review* | | | | |
| Viewed encounters | 110.4 (100.4) | 122.8 (167.0) | 81.7 (65.6) | 84.2 (59.6) |
| Edited encounters | 35.0 (24.5) | 34.8 (23.9) | 18.3 (16.4) | 17.9 (13.3) |
| Chart review actions | 158.4 (117.0) | 190.4 (137.4) | 85.6 (67.2) | 104.5 (78.5) |
| **Ordering Intensity** | | | | |
| *Ordering Sessions* | | | | |
| Ordering sessions | 19.5 (19.3) | 20.4 (18.8) | 13.6 (12.2) | 13.2 (12.3) |
| *Medications Modifications* | | | | |
| Medication list changes | 19.0 (26.8) | 24.0 (31.0) | 13.2 (14.9) | 12.6 (15.0) |
| Medication orders | 18.0 (25.5) | 22.0 (29.3) | 12.5 (14.1) | 12.1 (14.3) |
| Patients with medication orders | 8.0 (10.7) | 9.0 (11.3) | 5.4 (5.2) | 5.1 (5.3) |
| **Clinical Communication** | | | | |
| *Patient-specific Messaging* | | | | |
| Patient-message sent | 1.3 (3.5) | 2.7 (25.0) | 3.3 (6.6) | 4.6 (6.3) |
| Patients with a patient-message sent | 1.1 (3.1) | 2.4 (24.8) | 2.9 (6.0) | 4.0 (5.4) |
| Patient-messages viewed | 2.3 (5.2) | 5.1 (8.3) | 5.0 (6.9) | 7.3 (8.6) |
| Patients with a patient-message viewed | 1.9 (4.0) | 4.2 (6.4) | 3.9 (5.1) | 5.7 (6.4) |
| *Asynchronous Messaging* | | | | |
| In Basket messages created | 41.2 (42.6) | 42.0 (40.8) | 18.7 (15.5) | 17.8 (14.1) |
| In Basket messages viewed | 101.1 (101.0) | 114.2 (106.0) | 59.4 (60.6) | 66.1 (75.8) |

**Table S4**. Changes in physician EHR time across telemedicine encounter modalities. Values shown are average EHR time (*m*, SD) per physician-week, normalized per 8 patient scheduled hours (PSHs). F2F, face-to-face; TM, telemedicine.

|  | **WashU** | | | | **UCSF** | | | |
| --- | --- | --- | --- | --- | --- | --- | --- | --- |
| **Time-based Measures (per Provider-Week)** | **All F2F** | **Mixed (≤50%)** | **Mixed (>50%)** | **All TM** | **All F2F** | **Mixed (≤50%)** | **Mixed (>50%)** | **All TM** |
| EHR-Time_8_ | 9.8 (5.9) | 10.7 (5.4) | 13.9 (6.6) | 15.5 (7.0) | 6.0 (5.5) | 10.4 (6.8) | 12.7 (6.6) | 13.5 (7.5) |
| WOW_8_ | 4.4 (4.8) | 4.3 (4.5) | 6.7 (5.7) | 5.7 (6.0) | 3.3 (4.0) | 5.4 (5.2) | 6.2 (5.6) | 7.4 (6.3) |
| Note-Time_8_ | 6.8 (5.0) | 7.6 (4.7) | 10.9 (6.0) | 12.8 (6.1) | 3.9 (4.4) | 7.3 (5.6) | 9.7 (5.6) | 10.4 (6.4) |
| WOW-Note-Time_8_ | 2.8 (3.6) | 2.7 (3.3) | 4.7 (4.5) | 4.0 (4.6) | 1.9 (2.9) | 3.3 (3.8) | 4.1 (4.1) | 4.9 (5.0) |
| MOB_8_ | 0.4 (0.8) | 0.3 (0.6) | 0.3 (0.6) | 0.1 (0.3) | 0.2 (0.5) | 0.2 (0.4) | 0.3 (0.6) | 0.3 (0.7) |

**Table S5**. Changes in counts of EHR tasks across telemedicine encounter modalities. Values shown are average counts of EHR activity (*m*, SD) per physician-day. F2F, face-to-face; TM, telemedicine.

|  | **WashU** | | | | **UCSF** | | | |
| --- | --- | --- | --- | --- | --- | --- | --- | --- |
|  | **All F2F** | **Mixed (≤50%)** | **Mixed (>50%)** | **All TM** | **All F2F** | **Mixed (≤50%)** | **Mixed (>50%)** | **All TM** |
| **Information Seeking** | | | | | | | | |
| Outside record views | 4.0 (7.0) | 6.0 (8.7) | 3.0 (5.9) | 2.3 (5.9) | 4.1 (7.5) | 7.4 (11.0) | 11.8 (14.6) | 7.3 (11.5) |
| Patients with outside record views | 1.6 (2.4) | 2.4 (3.0) | 1.2 (2.0) | 0.8 (1.6) | 1.3 (1.7) | 2.3 (2.3) | 3.2 (2.7) | 2.1 (2.1) |
| Viewed encounters | 113.8 (188.8) | 155.1 (89.9) | 107.4 (69.3) | 73.9 (55.9) | 73.1 (58.0) | 98.7 (57.6) | 92.1 (55.7) | 77.0 (60.9) |
| Edited encounters | 33.1 (22.4) | 42.1 (26.1) | 27.4 (24.4) | 17.0 (18.5) | 16.9 (13.5) | 21.9 (13.7) | 18.6 (12.3) | 14.7 (11.8) |
| Chart review actions | 176.0 (125.1) | 246.5 (158.5) | 148.4 (108.4) | 86.6 (74.2) | 86.1 (69.9) | 130.3 (80.8) | 130.1 (79.9) | 87.2 (73.4) |
| **Ordering Intensity** | | | | | | | | |
| Ordering sessions | 18.2 (17.5) | 28.6 (20.8) | 15.4 (13.4) | 7.3 (8.2) | 11.0 (11.7) | 18.6 (14.0) | 13.5 (10.5) | 9.6 (9.2) |
| Medication list changes | 20.0 (28.7) | 36.0 (35.4) | 23.0 (23.9) | 13.0 (16.8) | 9.9 (13.6) | 17.8 (17.4) | 11.5 (13.2) | 10.2 (12.6) |
| Medication orders | 19.0 (27.1) | 34.0 (33.4) | 21.0 (22.5) | 12.0 (15.7) | 9.5 (13.0) | 17.1 (16.6) | 11.0 (12.5) | 9.6 (11.8) |
| Patients with medication orders | 8.0 (10.6) | 14.0 (12.6) | 9.0 (9.2) | 5.0 (5.9) | 4.1 (4.9) | 7.1 (6.0) | 4.8 (4.7) | 4.1 (4.5) |
| **Clinical Communication** | | | | | | | | |
| Patient-message sent | 2.2 (28.2) | 4.4 (15.8) | 2.7 (5.8) | 2.0 (4.4) | 3.5 (6.0) | 5.6 (6.7) | 4.1 (5.5) | 4.5 (6.1) |
| Patients with a patient-message sent | 2.0 (28.1) | 3.8 (15.1) | 2.2 (4.4) | 1.7 (3.4) | 3.1 (5.2) | 4.9 (5.7) | 3.6 (4.7) | 4.0 (5.2) |
| Patient-messages viewed | 4.0 (6.7) | 8.3 (11.1) | 6.9 (10.4) | 4.4 (7.3) | 5.8 (8.1) | 8.9 (9.4) | 7.2 (7.7) | 7.1 (8.2) |
| Patients with a patient-message viewed | 3.3 (5.3) | 6.8 (8.4) | 5.6 (8.0) | 3.5 (5.6) | 4.6 (6.2) | 6.9 (6.9) | 5.5 (5.7) | 5.4 (6.0) |
| In Basket messages created | 36.2 (36.9) | 62.2 (46.8) | 33.3 (29.7) | 18.5 (18.9) | 14.2 (12.8) | 20.2 (14.5) | 20.2 (14.3) | 17.9 (14.1) |
| In Basket messages viewed | 103.0 (97.8) | 153.3 (121.0) | 95.4 (95.8) | 65.9 (75.3) | 57.4 (79.0) | 79.5 (71.5) | 65.6 (68.5) | 60.5 (77.3) |

**Table S6**. Association between telemedicine encounter modalities and physician EHR use. Values shown are effect sizes (*β*, 95% CI). TM, telemedicine. Physician-weeks or -days consisting of entirely face-to-face encounters were used as the reference category in the multivariable regression model. (*: p < .05)

|  | WashU | | | UCSF | | |
| --- | --- | --- | --- | --- | --- | --- |
|  | Mixed (≤50%), *β* (95% CI) | Mixed (>50%), *β* (95% CI) | All TM, *β* (95% CI) | Mixed (≤50%), *β* (95% CI) | Mixed (>50%), *β* (95% CI) | All TM, *β* (95% CI) |
| **Time-based Measures (per Provider-Week)** | | | | | | |
| EHR-Time_8_ | 0.2* (0.0–0.4) | 0.3 (-0.6–1.2) | 3.1* (1.3–5.0) | 0.3 (-0.0–0.5) | 0.6* (0.2–1.0) | 1.4* (0.8–1.9) |
| WOW_8_ | 0.1 (-0.0–0.3) | -0.2 (-1.1–0.8) | 1.0 (-0.4–2.4) | 0.0 (-0.3–0.2) | -0.3 (-0.6–-0.1) | 0.2 (-0.2–0.7) |
| Note-Time_8_ | 0.2* (0.1–0.4) | 0.3 (-0.5–1.1) | 2.7* (1.1–4.4) | 0.3* (0.1–0.5) | 0.8* (0.5–1.1) | 1.4* (1.0–1.9) |
| WOW-Note-Time_8_ | 0.1 (-0.0–0.2) | -0.2 (-1.0–0.5) | 0.7 (-0.3–1.8) | 0.0 (-0.1–0.2) | -0.1 (-0.3–0.2) | 0.2 (-0.1–0.5) |
| MOB_8_ | -0.0 (-0.0–0.0) | 0.0 (-0.1–0.1) | -0.1 (-0.2–0.0) | -0.0 (-0.0–0.0) | -0.0 (-0.0–0.0) | -0.0 (-0.0–0.0) |
| **Frequency-based Measures (per Provider-Day)** | | | | | | |
| *Information Seeking* | | | | | | |
| Outside record views | -0.0 (-0.1–0.1) | -0.5* (-1.0–-0.0) | -1.0* (-1.5–-0.6) | 0.8* (0.5–1.1) | 2.0* (1.4–2.5) | 0.8* (0.5–1.2) |
| Patients with outside record views | 0.0 (-0.0–0.1) | -0.1 (-0.3–0.0) | -0.3* (-0.5–-0.1) | 0.2* (0.1–0.2) | 0.5* (0.3–0.6) | 0.1* (0.1–0.2) |
| Viewed encounters | 0.2 (-2.4–2.8) | 1.7 (-3.3–6.7) | -10.9* (-20.1–-1.6) | 3.6* (1.8–5.3) | 5.1* (2.6–7.7) | 0.7 (-1.5–3.0) |
| Edited encounters | 0.2 (-0.2–0.6) | 0.1 (-0.9–1.2) | -2.4 (-4.9–0.2) | 0.5* (0.1–0.9) | 0.4 (-0.1–0.9) | -0.4*(-0.8–-0.0) |
| Chart review actions | 2.0 (-0.2–4.1) | -0.2 (-9.6–9.3) | -15.3* (-30.1–-0.5) | 8.6* (5.9–11.3) | 9.5* (5.8–13.2) | -3.0* (-5.8–-0.2) |
| *Ordering Intensity* | | | | | | |
| Ordering sessions | 0.4* (0.1–0.7) | -0.4 (-1.3–0.5) | -2.6* (-5.1–-0.0) | 1.0* (0.6–1.5) | -0.6 (-1.3–0.1) | -2.8* (-3.3–-2.3) |
| Medication list changes | 1.1* (0.6–1.5) | 1.3* (0.0–2.5) | -2.3 (-5.3–0.6) | 0.7* (0.2–1.1) | -0.5 (-1.2–0.1) | -1.8* (-2.4–-1.3) |
| Medication orders | 1.0* (0.5–1.4) | 1.1 (-0.1–2.3) | -2.2 (-5.0–0.6) | 0.6* (0.2–1.1) | -0.5 (-1.2–0.1) | -1.8* (-2.3–-1.3) |
| Patients with medication orders | 0.3* (0.2–0.5) | 0.2 (-0.3–0.6) | -0.8 (-2.0–0.3) | 0.3* (0.1–0.4) | -0.1 (-0.4–0.1) | -0.6* (-0.8–-0.4) |
| *Clinical Communication* | | | | | | |
| Patient-message sent | 0.1 (-0.1–0.2) | 0.1 (-0.3–0.4) | -0.3 (-0.7–0.2) | -0.0 (-0.2–0.1) | 0.2 (-0.1–0.4) | 0.2* (0.0–0.5) |
| Patients with a patient-message sent | 0.1 (-0.1–0.2) | 0.1 (-0.3–0.4) | -0.3 (-0.7–0.2) | -0.0 (-0.1–0.1) | 0.2 (-0.0–0.3) | 0.2* (0.0–0.4) |
| Patient-messages viewed | 0.2* (0.0–0.4) | 0.4 (-0.0–0.9) | -0.3 (-1.0–0.3) | 0.1 (-0.1–0.3) | 0.1 (-0.2–0.4) | 0.1 (-0.2–0.3) |
| Patients with a patient-message viewed | 0.2* (0.0–0.3) | 0.3 (-0.0–0.7) | -0.3 (-0.9–0.2) | 0.1 (-0.1–0.2) | 0.1 (-0.1–0.4) | 0.0 (-0.1–0.2) |
| In Basket messages created | 0.0 (-0.7–0.7) | -3.1* (-4.8–-1.4) | -7.4* (-13.3–-1.5) | 0.6* (0.2–1.0) | 1.9* (1.2–2.5) | 0.8* (0.2–1.3) |
| In Basket messages viewed | 0.6 (-1.2–2.4) | -1.5 (-7.4–4.4) | -12.3* (-24.3–-0.2) | -0.6 (-2.4–1.3) | 0.1 (-2.6–2.7) | -0.7 (-2.9–1.6) |
